# Birds of a song sing together: adaptive choices in the use of shared vocalizations that indicate age

**DOI:** 10.3389/fpsyg.2025.1567261

**Published:** 2025-09-10

**Authors:** Francisco R. Magdaleno, Devin Zaratzian, Ginnes Paladini-Colon, Anya R. Jessum, Adrian L. O’Loghlen, Stephen I. Rothstein

**Affiliations:** ^1^Department of Psychology, California State University Channel Islands, Camarillo, CA, United States; ^2^Department of Ecology Evolution & Marine Biology, University of California, Santa Barbara, Santa Barbara, CA, United States

**Keywords:** sexual selection, vocal learning, song usage, age, oscines, cowbirds, vocal functional flexibility, cultural evolution

## Abstract

The study of non-human animals that are vocal learning, which is the ability to alter the structure of vocalizations, has provided important insights into our understanding of the evolution and development of vocal communication and language. Of particular interest is vocal convergence where individuals learn the vocalizations shared by their social group. Although the development of shared vocalizations has received extensive study across vocal learning species, less attention has been given to the use of these learned signals, especially whether individuals stress the expression of vocalizations that signal high status and deemphasize those that do not. Previous field and captivity studies of Brown-headed Cowbirds (*Molothrus ater*) show that males 2 years old or older have greater levels of song type sharing than 1 year old males and that females are more sexually stimulated by shared song types than by the non-shared types that occur mostly in the repertoires of yearlings. In this study, we observed males at least 2 years old singing in experimentally paired matches with another male or female conspecific to determine whether they preferentially used some song types in their repertoires and whether preferences were based on shared songs that indicate male age. Data on over 5,000 songs by 12 males showed that males did not use song types randomly. The most frequently used song types directed to both males and females were types shared with the most other males. This was the case whether males or song types were the unit of analysis, clearly demonstrating that this species emphasizes vocalizations that signal high quality (an age of 2 years or more) and therefore optimizes the use of what it has learned. These results support the hypothesis that selection can favor stable presentation of shared vocalizations across social contexts because age-revealing information in vocalizations that require learning significantly improves the accuracy of age assessment, which in many species is typically based on maturational changes that occur with aging.

## 1 Introduction

The comparative study of learned communicative traits in non-human animals is essential for our understanding of the evolution of language and vocal communication in general (Fitch, 2017; Jarvis, 2019). Non-human animals have importantly provided insights into the sexually selected bases (Beecher and Brenowitz, 2005; Catchpole and Slater, 2003) and development (Doupe and Kuhl, 1999; Gervain and Mehler, 2010) of vocal learning, which is the relatively rare ability to alter the acoustic properties of vocalizations in response to experience (Janik and Knörnschild, 2021; Martins and Boeckx, 2020; Vernes et al., 2021). Of particular interest are vocal learning species that copy the vocalizations of conspecifics in what is referred to as vocal convergence (Janik and Knörnschild, 2021). A bias toward learning the vocalizations shared by a social group is one of the most well-supported mechanisms of transmission in cultural evolutionary theory (Lachlan et al., 2018; Whiten, 2019; Williams, 2021) and is considered to provide humans and other animals various social benefits (Harrison et al., 2024). Humans learn their native language down to the local dialect of their social group (Cohen, 2012), and during speech, flexibility is a core feature with drastic modulation of lexical, prosodic, and phonetic vocal features (Ruch et al., 2018; Magdaleno et al., 2024) used to fulfill different communicative needs, including short-term vocal accommodation (Ruch et al., 2018), based on the addressee(s) and social context (e.g., conversation with a close friend vs. an unfamiliar individual during a job interview). Although vocal learning during development and the acquisition of shared vocalizations in the laboratory and in nature has received extensive study (Catchpole and Slater, 2003), less attention has been given to the use of learned vocal repertoires, especially whether animals stress the expression of signals within their repertoires that signal high status and deemphasize those that do not. Vocal convergence in vocal learners is common, but is it also common for individuals to use the learned phonological signals in their repertoires in an adaptive manner? We address this question in the present study by determining whether males in a well-studied songbird species overuse those song types in their repertoires that are most effective in stimulating female sexual responses, namely those types widely shared in their local population.

Songbirds have been one of the most studied vocal learners other than humans (Aplin, 2019; Williams, 2021). Unlike other vocal learning species, such as dolphins and most bats, vocalizations in songbirds are a predominantly sexually selected trait (Gil and Gahr, 2002; Nowicki and Searcy, 2014; Vernes and Wilkinson, 2020), making them suitable species for studying the use of learned traits that function adaptively as status or quality signals in intersexual and/or intrasexual interactions. Such features, which are called indicator traits, are widespread across animal groups (Johansson and Jones, 2007; Crothers and Cummings, 2015; Jordania, 2021; Locke, 2017; Conroy-Beam and Buss, 2019). Morphological indicator traits include skin coloration in the Strawberry Poison Frog (*Oophaga pumilio*; Crothers and Cummings, 2015), tail length in peacocks (*Pavo cristatus*; Jordania, 2021), and diet-dependent and disease related plumage coloration in the House Finch (*Haemorhous mexicanus*; Hill, 1991; Zahn and Rothstein, 1999). Vocal cues and signals can also be indicator traits that can be expressed variably. For example, roaring in Scottish Red Deer (*Cervus elaphus*) is an honest indicator of body size and fighting ability (Clutton-Brock and Albon, 1979) and time spent singing in birds is an indicator of fitness (Gil and Gahr, 2002). But all of these aforementioned indicator traits are not the result of learning unlike the vocalizations of a significant number of songbirds that communicate a singer’s quality via variation in repertoire size (i.e., number of learned phonological signals), repertoire content (i.e., the type of learned phonological signals), and quality of song performance (Kroodsma, 1979; Mountjoy and Lemon, 1996; Hasselquist et al., 1996; Buchanan and Catchpole, 1997; Nowicki et al., 1998, 2002; Beecher and Brenowitz, 2005; Catchpole and Slater, 2003; Dadwal and Bhatt, 2017; dos Santos et al., 2018; Robinson and Creanza, 2019; but see, Byers and Kroodsma, 2009; Soma and Garamszegi, 2011; Verner, 1975).

Although there are extensive studies on morphological and behavioral indicator traits, there is limited research (but see Beecher and Brenowitz, 2005; Kiefer et al., 2011; Lapierre et al., 2011) on the extent to which animals other than humans make adaptive choices in their use of learned vocal repertoires. The relative adaptiveness of different learned vocalizations is especially well understood in the Brown-headed Cowbird (hereafter cowbird), a songbird with a repertoire averaging 5 (range 2–8) learned song types called “perched songs.” These are used during the breeding season in courtship (Friedmann, 1929; Dufty, 1985; O'Loghlen and Rothstein, 1995, 2003; Hosoi et al., 2005) and in male–male competition (Rothstein et al., 1988). Perched song is almost always given while perched in contrast to another learned male vocalization, the flight whistle, which is usually given in flight and which is acoustically distinct from perched song (Friedmann, 1929; Rothstein et al., 1988). This study does not deal with flight whistle data which are dealt with in other papers (Rothstein and Fleischer, 1987; O'Loghlen and Rothstein, 1993, 2002, 2003) and all further mention herein of songs refer to perched songs unless otherwise noted.

Playback experiments in captivity show that larger song repertoires and locally shared song types elicit stronger sexual responses from female cowbirds (O'Loghlen and Rothstein, 1995, 2002, 2003; Hosoi et al., 2005). Additionally, support for the significance of repertoire size and content also comes from ontogenetic studies in nature (O'Loghlen and Rothstein, 1993; O'Loghlen, 1995) showing that males (i.e., 1 year old adult males in their first breeding season) alter their song repertoires after their first breeding season but before the second by enlarging their repertoires and replacing some or all of their unshared song types with types that are widely shared with local males at least 2 years old. These first- to second-year changes that occur in nature have been replicated in captivity by experimentally altering the song types yearling males experience (O'Loghlen and Rothstein, 2010a). Although the non-shared song types of yearlings do not match any song types found in the repertoires of other local cowbirds, they have a song structure typical for the species and appear to be normal cowbird song types when compared to songs sung by older males. Yearlings are sexually mature but are less likely to obtain copulations than older males (Yokel, 1989), which is a likely result, at least in part, of their paucity of shared song types. Songs vary spatially but song sharing in local natural populations is high and especially so among adult males 2 years old or older who have repertoires consisting mostly of song types shared with other local adults (Dufty, 1985; O'Loghlen and Rothstein, 1993, 2002). In addition, cowbirds typically have one version of their other learned vocalization, flight whistles, which vary spatially in well-defined dialects and also indicate age (ASY versus SY; O'Loghlen and Rothstein, 1993, 2002).

The shift in song repertoires between a male cowbird’s first and second breeding season suggests that there is a benefit to having a repertoire of locally shared song types, which in turn leads to the hypothesis that these repertoires indicate whether a male is a yearling or an older bird that has survived for 2 or more years (O'Loghlen and Rothstein, 2003). Surviving the first year of life in the wild is not trivial given the well-documented high mortality rates during this period in various animal groups (Sæther, 1989; Liker and Székely, 2005; Ricklefs et al., 2011), including cowbirds (Fankhauser, 1971; Johnson et al., 1980). Because older individuals have demonstrated their ability to survive longer, age is generally considered to be an indicator of genetic quality and a likely factor in female mate choice in cowbirds (Rothstein and Fleischer, 1987; O'Loghlen and Rothstein, 2003), other bird species (Schmoll et al., 2007; Dickinson, 2001; Lapierre et al., 2011; Kochvar et al., 2022; Schlicht and Kempenaers, 2023), and many other animals (Manning, 1985; Kokko, 1998; Brooks and Kemp, 2001; Jacot et al., 2007; Conroy-Beam and Buss, 2019). Although social skills that males develop may also contribute to male mating success (West et al., 1981; Gros-Louis et al., 2009), the predominant factors that likely affect female mate choice in this brood parasitic species in nature are traits that indicate genetic quality, which correlates with male age (Kokko, 1998; Manning, 1985), because the only direct contribution to reproduction that female cowbirds are known to acquire from males is genetic (i.e., their sperm). Cowbirds, as brood parasites, do not provide parental care and there is no evidence that males in the wild provide any services to females with whom they mate (Yokel and Rothstein, 1991).

Despite the evidence cited above that shared song types are an indicator trait that females can use to assess male age and indirectly relative male genetic quality, it is unknown if male cowbirds overuse (i.e., use more frequently) the widely shared song types in their repertoires that indicate older age and underuse unshared types. Recent work on cowbird song type use in captivity (Magdaleno et al., 2024) found that males overuse some song types in their repertoires and underuse others, but neither differences in song duration nor whether a song type was shared differentiated overused song types from underused song types. That study, however, could not address the importance of shared songs as song sharing among the subjects was much lower than in local wild populations because the study mixed birds from two distant localities and included males with a range of early experience regimens (unlike the present study). Similarly, while previous captivity-based studies (see King and West, 1977; West et al., 1983; King and West, 1989; West et al., 1996; King et al., 2005) assessed the potency male cowbird songs have in eliciting female sexual responses, they did not test for the importance of locally shared song types.

The present study used 12 males (*M. a. obscurous*) caught as adults 2 years old or older (i.e., ASY or after second year) in one local area in Santa Barbara. These males had undergone a normal ontogeny in nature which was completed by the time they were captured as males in nature are not known to change song repertoires after their second year (Dufty, 1985). We predicted that males are more likely to sing shared rather than non-shared song types when singing to males or females and the most frequently sung song types will be those that are shared with most other males. This prediction was tested with both individuals and song types as the unit of analysis by recording males while they were matched with another male or female and isolated from other cowbirds. Recordings of over 5,000 songs revealed every male’s complete song type repertoire, how often males used each song type in both male- and female-directed social contexts, and whether there was a relationship between level of song type sharing and song type use.

## 2 Methods

### 2.1 Subjects

Two categories of birds, subjects and targets, were involved. Subjects were 12 ASY males whose recorded song types are the focus of this paper, and target birds (either ASY males or females) were the individuals to whom subjects directed their songs as in Magdaleno et al. (2024). Unlike most songbirds, many and possibly most songs by male cowbirds are directed at a conspecific male or female a meter or less away (Friedmann, 1929; Magdaleno et al., 2024) by a male facing the individual as a song begins. So the intended target of “directed song” is clear in this species. All subject males were aged in the field as ASYs upon capture based on their underwing coverts which are all black in ASYs and contain brown feathers in SYs (Selander and Giller, 1960; Ortega et al., 1996). Three of the 12 subjects were captured in 2006 in Santa Barbara, California. They had been housed in an outdoor cage at the University of California, Santa Barbara (UCSB) aviary facility since their capture and served as models that other birds could copy vocally in a previous study (O'Loghlen and Rothstein, 2010a). The remaining 9 males were captured in 2009 in Santa Barbara and housed in cages within 5m of the 3 other males. Male target birds consisted of 11 randomly selected birds from the subject category, and female target birds were 2 ASY birds trapped in Mammoth Lakes, Mono County, California in 2005. All birds were housed under natural photoperiod with food (Mazuri Small Bird Maintenance kibble supplemented with mealworms) and water provided *ad libitum*. The males and female birds were housed separately in adjacent cages (i.e., 12 males in 1 cage and 2 females in another) that were each 1.2 × 2.7 × 6.0 m.

### 2.2 Recording procedures

In preparation for the recordings in 2011, subject and target birds were removed from the outdoor pens and placed into individual cages (46 × 27 × 27 cm) that had a perch running its length as well as food and water provided *ad libitum*. These cages were placed indoor inside acoustically isolated chambers (interior dimensions of 61 × 33 × 38 cm; O’Loghlen and Rothstein, 2010b) at the UCSB Aviary where birds acclimated for 48 h before being recorded. Interior lighting was provided by a 12V DC fluorescent light controlled by an outdoor photocell, keeping a natural photoperiod. Temperature controlled air was supplied to the interior of the isolation chambers with an air pump. Subject males were rotated into isolation throughout the 2-month recording period (April 12–June 14, 2011) with only 4 subjects and 2 targets in isolation at any one time. Once placed in isolation, subjects and targets were acoustically isolated except during the recording sessions.

In the recording sessions, always between 0730 and 1100h, a subject male in his individual cage was moved into a recording chamber (61 × 71 × 61cm) lined with noise dampening acoustic foam (2″ pyramid Sonitec™, foamorder.com, San Francisco, CA). A target male or female in its individual cage was then immediately placed into the recording chamber next to the subject’s cage. Light was provided by two 25cm long 110V fluorescent lights mounted on the ceiling. A Sennheiser ME80 microphone was on a tripod and suspended above and directly in between the subject and target cages with the microphone facing downward evenly spaced between both cages. While perched, the subject and target were 15-20cm from the microphone. Subject and target cage walls were 3cm apart, which allowed the birds to see and hear each other. The distance between subjects and targets (30-40cm) is consistent with the distance over which perched song displays are directed to conspecifics in the wild (Rothstein et al., 1988) and in aviary settings (Magdaleno et al., 2022). Additionally, 3 webcams were placed inside the chamber to monitor the behavior of the subject and target. The webcams and Sennheiser microphone were connected to 2 computers, one of which displayed video from all three webcams while the second recorded audio.

Subjects underwent multiple recording sessions with the goal of recording 200 songs to each target sex by each subject (see Table 1 for total songs recorded from each subject). Because subjects occasionally sang immediately after the target was introduced (though this was rare), recording began before the target was placed into the recording chamber. Most subjects started to sing within the first 5 min of the recording chamber lid closing, and all sang to females within the first 20 min. In some male-to-male sessions, the subject did not sing within the first 20 min, and these recording sessions were terminated. On the next recording day, a subject that did not sing to a male was exposed to a different target male. Recording sessions were capped at either 100 songs or 1 h of recording. Subjects were limited to 1 recording session per day. Each subject had at least 2 recording sessions to each target sex (i.e., each subject was recorded for a minimum of 4 days total). Some birds required more than 2 sessions to reach the goal of 200 songs, and the maximum number of bouts to a target sex was 8. Subjects were returned to their original flight cages after recording goals were met. Of the 2 females that served as targets, 1 was a target for all 12 male subjects and 1 a target for 4 males. Among the 9 target males, 6 served as targets for 1 subject male each, 2 as a target for 4 subject males, and 1 as a target for 2 subject males. A male target sang to the subject in only 8 out of more than 50 bouts, and these 8 bouts were not used in the analysis.

**Table 1:** The song type repertoires and total number of songs each male sang to females and males.

Subject	Repertoire Size	Song Types in Repertoire	Total Songs Directed to Females	Total Songs Directed to Males
CBM1	5	A, C, D, E, F	276	223
CBM2	5	A, B, C, F, U*	218	220
CBM3	4	A, C, E, F	164	245
CBM4	6	A, B, C, D, F, G	210	275
CBM5	4	A, C, E, I*	208	196
CBM6	4	A, C, F, G	217	230
CBM7	4	A, B, C, F	219	209
CBM8	5	A, B, C, D, F	225	254
CBM9	4	A, B, C, F	200	286
CBM10	5	A, B, C, D, W*	213	241
CBM11	5	A, B, C, D, F	226	241
CBM12	3	A, C, H*	266	251

Each letter represents the same song type across all individuals. Non-shared song types done by only 1 male have an (*).

**Figure 1:**
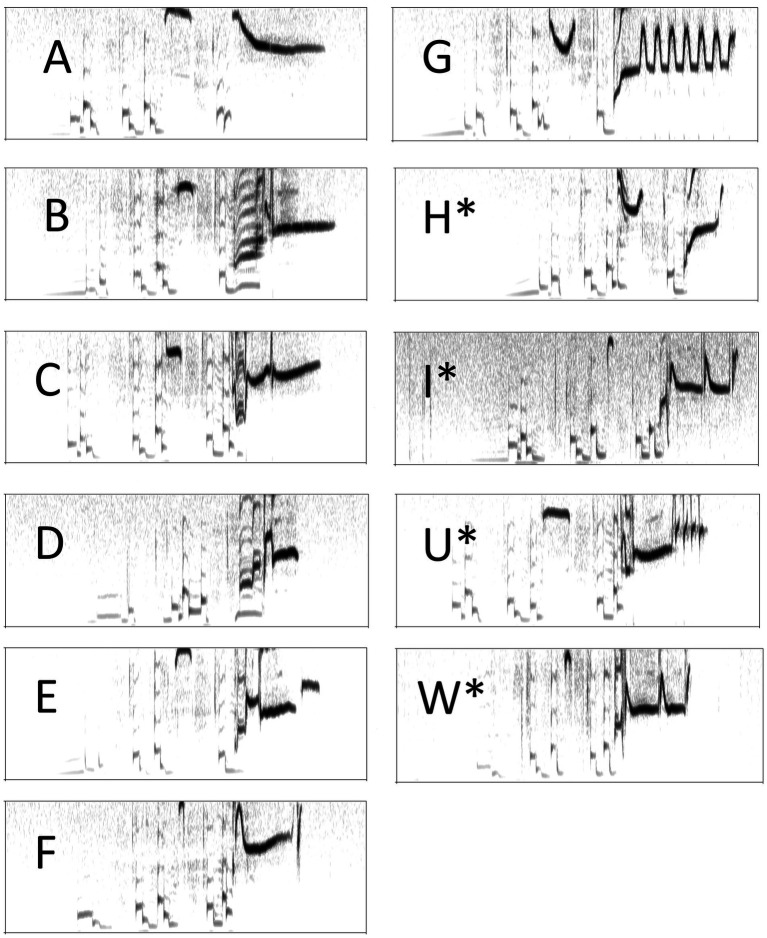
Sonograms of the 11 song types **(A–W)** produced by the 12 male subjects trapped in Santa Barbara County. Non-shared song types **(H–W)** done by only 1 male have an (*). All song time axes are 1.2 seconds in length (*x* axis) with a frequency range of 0-12,000 Hz (*y* axis). The recordings of each song type are provided in Supplementary material.

Song types are easily determined in cowbirds (King and West, 1989; Ronald et al., 2015; Gersick and White, 2018; Magdaleno et al., 2024) because the note cluster and whistle elements of a particular song type and their sequence are consistent within and across male repertoires (i.e., song types do not have intermediates & do not structurally change during the breeding season). The song repertoires of each male were first qualitatively classified by DZ by visually comparing all recorded songs (2,642 female-directed & 2,871 male-directed songs) to a song type catalog developed in previous studies of perched songs in the Santa Barbara area (Hosoi et al., 2005). Separately, FRM classified the song repertoires using the clear acetate method (Magdaleno et al., 2024) where each song recording was opened in Praat, V6.3.14 (Boersma, 2001; RRID:SCR_016564) and a selection window length set to 1.2 sec was placed around each song. All cowbird songs are ~1 sec in length, so a 1.2 sec window ensured all song types were analyzed as complete songs. The Paint Visible Spectrogram function with a picture grid set to 12 box units wide by 4 box units tall was performed on each song recording and its spectrogram was printed on transparent acetate. All songs were compared to all other songs by placing the acetate of one of the songs over another and aligning them to maximize overlap. Two songs were the same song type if each note cluster in the song type had the same number of notes with all of the notes overlapping at least partially and a 75-100% overlap of the whistle as in King and West (1989). This was performed until all the song recordings were categorized and tabulated based on song type and all the song types for each male were printed on a separate transparency. DZ counted Song Type I as being the same song type as Song Type W (Table 1), but this was incorrect because Song Type I has a different first note cluster than Song Type W (see Figure 1). There were 79 instances (43 female-directed & 36 male-directed) of Song Type I being produced by CBM5, who was the only male with this song type; therefore, the error rate in categorizing the song types among the two methods used by DZ and FRM was low at 0.014.

### 2.3 Analysis

Recording bouts for each subject were partitioned into male- and female-directed sets, and recordings from multiple days for an individual subject were combined to determine the subject’s repertoire and numbers of each song type he performed. For each target sex, Chi-square Goodness of Fit (GoF) tests (Magdaleno et al., 2024) were used to determine whether individuals deviated significantly from the expected results under a null hypothesis of random use, which predicted that each song type would be done T/n times where T equals the total number of songs recorded and n is the size of the male’s repertoire. This was completed separately for songs directed to males and to females as well as for male and female counts combined.

We next evaluated whether song type use was related to the extent to which a song type was shared in the captive population to test the prediction that favored song types were ones widely shared in the captive population. This was done by testing for a correlation between how often a subject performed each of his song types and how many males in the population had those types in their repertoires. This was statistically challenging because the observed repertoire sizes, 3–6, were too small to determine whether there were significant correlations for individual males. To overcome the low statistical power due to the males’ repertoire sizes, we calculated Pearson’s correlation coefficients for the number of times a male performed a song type and the number of males in the study population that had that song type in their repertoire. This was not to determine whether individual males showed a statistically significant trend, but only to test the null hypothesis that positive and negative correlations across all subjects are equally likely if there is no relationship between song type use and song type sharing. Population wide statistical significance could then be assessed by applying Sign tests (Magdaleno et al., 2024) to the number of males with positive versus negative coefficients. We also tested whether males changed their song type use in female- vs. male-direct contexts using the same approach with positive versus negative correlation coefficients and a Sign test across all males with positive correlation coefficients indicating similar or correlated song type use between contexts.

The above analysis assessed the relationship between males’ use of song types and the relative presence of those song types in the repertoires of all males by using individual males as the basic units of analysis. Likewise, it was possible to test this relationship using song types as the units of analysis. This was done by averaging the proportional performance for each song type across all subject males that had that song type in their repertoires. We then used these values to assess the correlation (Pearson’s correlation coefficient) between the average proportional use for each song type and the number of males in the population with that song type in their repertoire. In this case, the sample size was 11 because that is how many song types occurred in the study population. This analysis is not independent of the previous one but analyzes the data from another perspective. All Chi-square GoF, Pearson regression, and Sign tests were two-tailed and conducted using SPSS, V28.0 (IBM Corp, 2017; RRID: SCR_002865). This study was approved by the UCSB Institutional Animal Care and Use Committee (Protocol No. 185).

## 3 Results

Nine of the 12 subject males performed the targeted number (200) of female-directed songs to 1 target female while 3 performed them to 2 different females (average of 1.25 target females ± SE 0.131). Seven of the 12 subjects performed the targeted number of male-directed songs to 2 target males while 5 did so to 3 different target males (average of 2.42 target males ± SE 0.149). The song type repertoire size of each subject was identical for songs directed at male or female targets and averaged 4.5 song types (range: 3–6), which is consistent with previous reports of ASY repertoire sizes (O'Loghlen and Rothstein, 1993, 2002; Hosoi et al., 2005). Subjects produced 11 different song types, 7 of which were shared by at least 1 other male while 4 types (H, I, U, and W; Figure 1 and Table 1) were non-shared and produced by one male only. Among the 7 shared song types, 2 were produced by all 12 males with the remainder performed by 2–9 birds (Table 2).

**Table 2:** Song types by proportion of males with each song type and average frequency of use when directed to females and males.

Song type	Percent of males with song type (Number)	Average frequency of use when singing to females %	Average frequency of use when singing to males %
A	100% (12)	25%	22%
B	58.33% (7)	22%	22%
C	100% (12)	27%	33%
D	41.66% (5)	16%	13%
E	25% (3)	21%	24%
F	75% (9)	18%	17%
G	16.66% (2)	12%	5%
H*	8.33% (1)	21%	15%
I*	8.33% (1)	20%	18%
U*	8.33% (1)	2%	2%
W*	8.33% (1)	19%	18%

Non-shared song types done by only 1 male have an (*).

**Figure 2:**
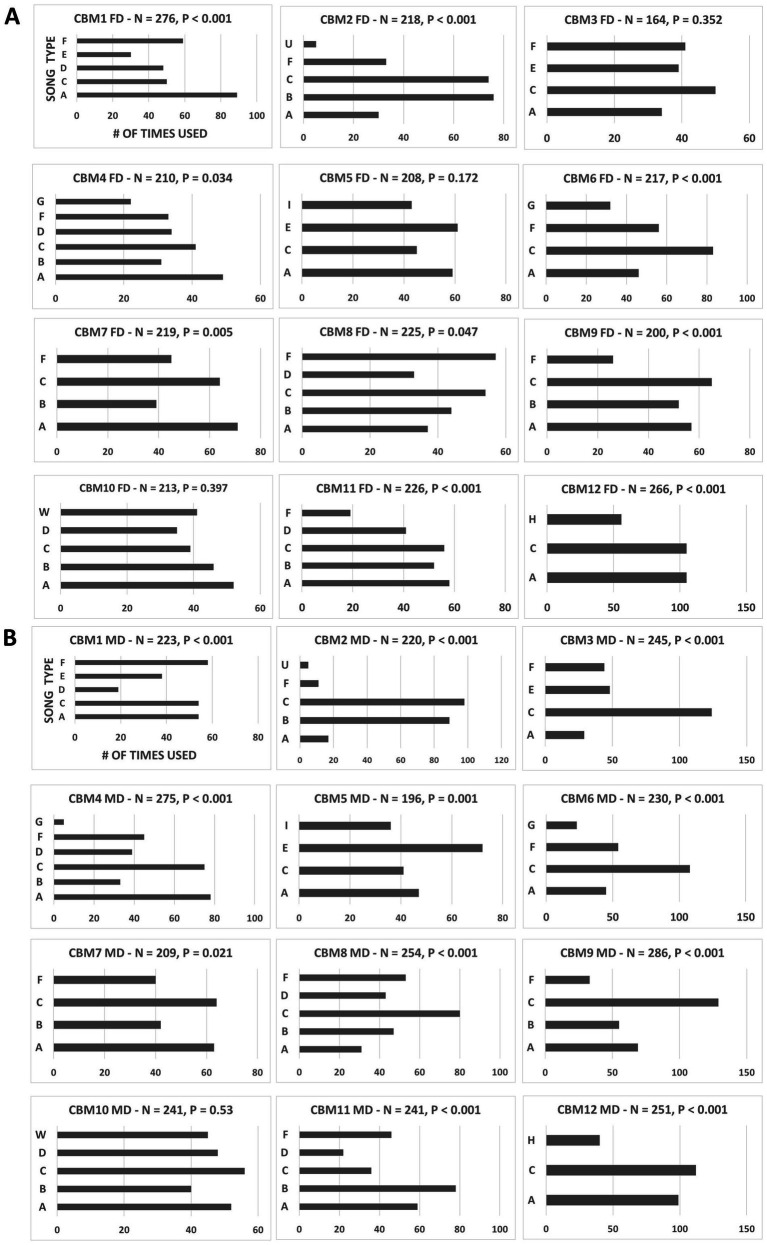
**(A)** Frequency of song type use by individual males when singing to females. Each box shows the frequency of song type use for each subject male, including total songs performed (N) and significance value for deviation from random use (Chi-square GoF test). **(B)** Frequency of song type use by individual males when singing to males. Each box shows the frequency of song type use for a subject male, including total songs performed (N) and significance value for deviation from random use (Chi-square GoF test).

Eleven of the 12 subjects showed a significant preference for certain song types in their repertoires when directing songs at target males (GoF test, 11 of 12 males, *P*’s ≤ 0.021; Figure 2). Similarly, 9 of the 12 subjects showed significantly non-random song type choices when singing to females (GoF test, 9 of 12 males, *P*’s ≤ 0.047; Figure 2B). And with female- and male-directed songs lumped and analyzed together, 11 of the 12 subjects showed significantly non-random song choices (GoF test, 11 of 12 males, *P*’s < 0.001). Furthermore, 11 of the 12 subjects showed a positive correlation between song type use when singing to females and to males (Sign test: *p* = 0.006). Thus, the same song types were favored by individual males whether they were singing to males or females.

**Figure 3:**
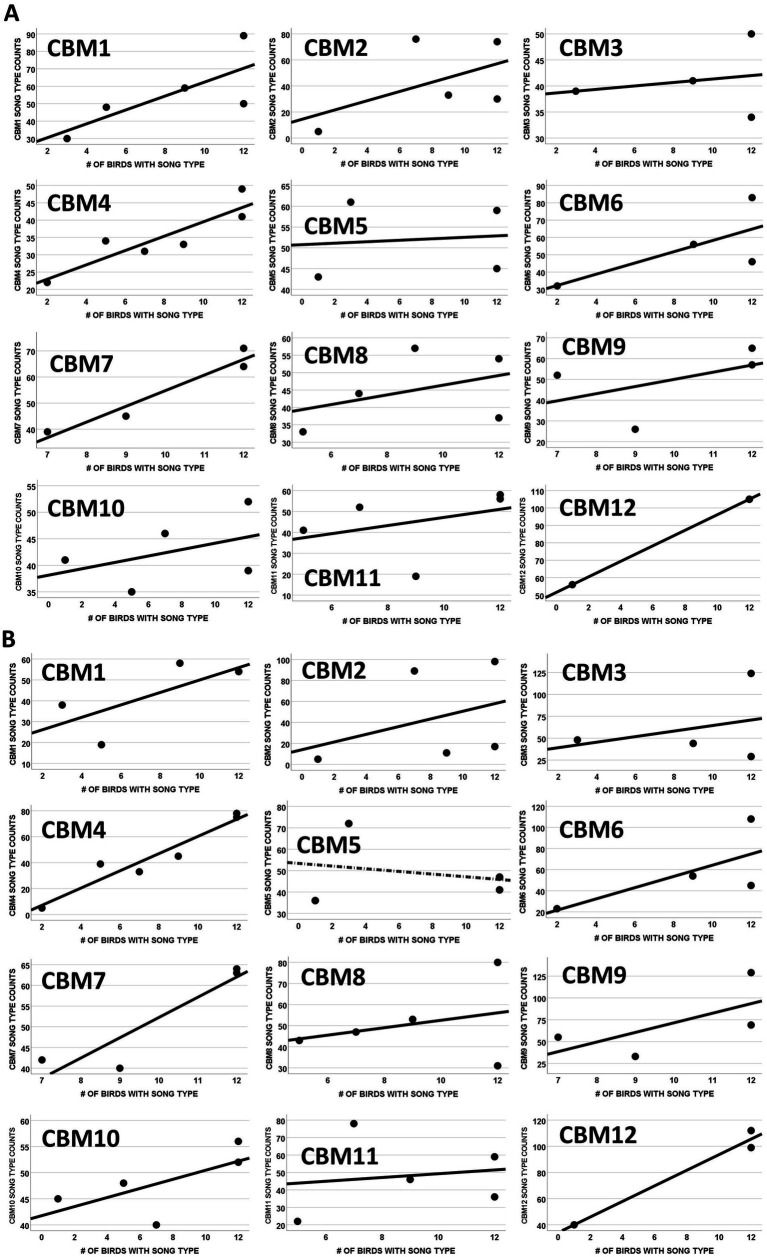
**(A)** Correlation of song type production by individual subject males for female-directed songs and the extent to which song types were shared among these males. The graphs show the relationship between the number of times each subject performed a song type when singing to a female and the number of subjects that shared that song type. All of the 12 males had positive slopes (*p* < 0.001, two-tailed Sign test), showing that males preferred to sing the most widely shared song types when singing to females. **(B)** Correlation of song type production by individual subject males for male-directed songs and the extent to which song types were shared among these males. The graphs show the relationship between the number of times each subject performed a song type when singing to a male and the number of subjects that shared that song type. A negative correlation is depicted with a checkered line. Eleven of the 12 males had positive slopes (*p* = 0.006, two-tailed Sign test), showing that males preferred to sing the most widely shared song types when singing to males.

Eleven of the 12 subjects showed a positive correlation between the number of times they used their song types when singing to male targets and the number of males in the study population that shared those song types in their repertoires (Sign test: *p* = 0.006; Figure 3A), and all 12 showed a positive correlation when performing to female targets (Sign test: *p* < 0.001; Figure 3B). This trend toward emphasizing highly shared song types can be seen most clearly with song types A and C, the only types present in the repertoires of all 12 subject males. A and C were the most frequently sung type for nearly all the males (9/12 subjects) for both male- and female-directed contexts (Figures 2A,B). This trend can also be seen with the song types (E, G, H, I, U, and W) present in the repertoires of the fewest males (3, 2, 1, 1, 1, and 1 respectively; Table 2), which were sung the least (Figures 2A,B and Table 2). In fact, 3 of the 4 non-shared song types were the least used song type by the male that sang that type (see song types H, I, and U in Figures 2A,B).

**Figure 4:**
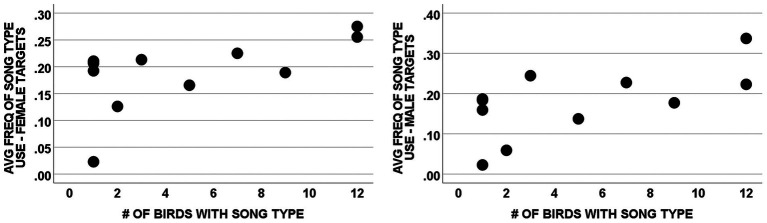
Correlation of average frequency of song type use and the extent to which song types were shared among subjects by social context. The graphs show the correlations between the average frequency with which a song type was produced by individuals with that type and the number of males with that type in their repertoire for songs directed at females [*r* (11) = 0.589, *p* = 0.057] and at males [*r*(11) = 0.626, *p* = 0.039]. Each point represents 1 of the 11 different song types given by the subject male. Two song types completely overlap in the graph for females (both *x* = 1, *y* = 0.21) and in the graph for males (both *x* = 1, *y* = 0.18).

As a further assessment of the relationship between song type use and song type sharing, we also analyzed our data using song types as units rather than from the perspective of individual males. We assessed the correlation between the mean of each male’s proportionate use of song types in his repertoire (averaged across all males that did that song type) and the number of males in the study population that had that type in their repertoire (Table 2 and Figure 4). These correlations were positive and significant for songs directed to males [*r*(11) = 0.626, *p* = 0.039; Figure 4] and positive and nearly significant for songs directed to females [*r*(11) = 0.589, *p* = 0.057; Figure 4]. This analysis has potential biases unlike our previous statistical tests. For example, there could be a potential confound due to the different repertoire sizes amongst subjects. If a male performed song types randomly, a male with a large repertoire would inherently have a lower proportionate use for each song type than a male with a small repertoire. Such a potential confound could produce the correlations shown in Figures 3A,B if rare song types were primarily present in the repertoires of males with large repertoires. However, this is not of concern here because 3 of the 4 unshared song types (I, U, and W; Table 2) were produced by males with average sized repertoires of 4 or 5 song types (Table 1), and the fourth unshared song type (H) was produced by the male with the smallest repertoire (3) in our sample, which would bias results against a significant correlation. Thus, the potential confound of varying repertoire sizes had no significant bearing on these results.

## 4 Discussion

Males showed non-random production of song types in both male- and female-directed singing. They used identical repertoires and favored the same song types in both contexts. Similarly, males of another cowbird subspecies (*M. a. ater*) recorded in large captive flocks with many possible recipients, including eavesdroppers, also had identical patterns of stable over and under use of song types across social contexts (Magdaleno et al., 2024). That previous study additionally showed that undirected songs with no target bird nearby, a context not tested in the present work, had overproduction of the same song types as in directed song. Similar song type use in both subspecies from distant localities (Pennsylvania and Indiana versus California) observed under different experimental conditions (flock with many different potential targets versus one-on-one paired matches with no other birds present) indicates that stable song type use across social contexts is a core characteristic of the communicative system in this vocal learning species. Over or under use of particular song types in the repertoires of individuals occurs in other songbirds such as Song Sparrows (*Melospiza melodia*; Lapierre et al., 2011), White-throated Sparrows (*Zonotrichia albicolis*, Otter et al., 2020), and Chestnut-sided Warblers (*Dendroica pensylvanica*; Byers et al., 2010).

The major novel finding of the present study is that the song types males favored were the most widely shared in the captive population. Assessments with both males and song types as the units of analysis showed that males overproduce their most highly shared song types whether they are singing to males or females. This finding demonstrates that males express an indicator trait optimally by overproducing song types that are most prevalent in older males and that elicit stronger female sexual responses than do non-shared songs (O'Loghlen and Rothstein, 1995, 2002, 2003). Previous work shows cowbirds tend to develop shared song types between their first and second breeding seasons (O'Loghlen and Rothstein, 1993, 2002), and the present study provides evidence of usage learning wherein cowbirds also learn to overproduce vocalizations in their repertoires that are most shared with other males.

### 4.1 A mechanism based on delayed vocal learning

Because ASY males sing highly shared local song types more often than less shared types (this study), yearling males may be more likely to hear and then learn those types than other types and to therefore produce those types as ASY males. This is a cycle that likely contributes to song stability because the more a particular song type is performed by ASY males, the greater the probability that yearling males will hear it and incorporate it into their repertoires. Future learning experiments in cowbirds will be needed to test if song type stability is due to frequency-based biases (song types heard the most) or model-based biases (preferentially learning song types performed by older males regardless of how often they are heard), the latter of which has been proposed to apply to cowbirds by Williams (2021). Whichever learning mechanism ends up being supported, it will nevertheless be based on the delayed vocal learning experienced by cowbirds due to the inadequate exposure juveniles have to adult vocalizations in their hatch year (O'Loghlen and Rothstein, 1993; O'Loghlen, 1995). Because young cowbirds are raised by heterospecific birds, they have much less exposure to conspecific songs early in development than do other songbirds. In addition, once female egg laying ends, cowbirds singing stops more abruptly than in non-parasitic songbirds because there is no benefit to maintaining pair bonds and site-based dominance (Rothstein and Fleischer, 1987; O'Loghlen and Rothstein, 1993). The extended 2-year song learning period is likely a consequence of this species’ parasitism and singing behavior. Although we do not have temporal data on perched song stability, field data (O'Loghlen et al., 2011, 2013) shows stability over almost 30 years in another cowbird song category, the flight whistle, which varies in small localized dialects.

If shared songs indicate high quality, the question then arises as to why males bother to ever sing unshared song types. In addition to shared song types, repertoire size is also an indicator of age in cowbirds as ASY males have larger repertoires than yearlings (O'Loghlen and Rothstein, 2002) and playback of a repertoire is more sexually stimulating to females than a single song type (Hosoi et al., 2005). If a male failed to display his total repertoire size by singing only shared song types, then that male would forgo signaling information that could additionally reveal age and might experience decreased mating success (see below). Similarly, if a male used only a subset of his repertoire when singing to males and a different subset to females, he would be forgoing the presentation of repertoire size. Overall, our study shows that males appear to make optimal use of their repertoires by using all song types when singing to male and female conspecifics, revealing repertoire size while also emphasizing shared song types that indicate that they are at least 2 years old and therefore likely of higher average genetic quality than yearlings.

### 4.2 Age assessment and the evolution of vocal functional flexibility

Male cowbird song directed to males serves a completely different function than song directed to females – aggression/threat versus courtship, respectively (Rothstein et al., 1988; Magdaleno et al., 2022). How then are different illocutions communicated when males use the same song types while singing to male vs. female conspecifics? Cowbirds accomplish this via modulation of the visual display that accompanies their songs as well as through changes to the prosodic qualities of song. The visual display done simultaneously with male song is an elaborate “wing-spread” display that can involve some or all of the following features each of which can vary in intensity: feather puffing, wing spreading and pumping, bowing, tail cocking, and bill wiping. The displays accompanying male-directed songs include more of these features and with more intense movements than songs directed to females (O’Loghlen and Rothstein, 2010b,c; Magdaleno et al., 2022). Cowbirds also modify the prosodic features (e.g., tone of voice) of a song type when singing to males vs. females (Ronald et al., 2015; Gersick and White, 2018). Therefore, the same song types can convey different illocutions (threaten to attempt to repel a male vs. seek to attract a female) in these two contexts through variation in the accompanying multi-modal features of song. Using the same vocalization to serve different illocutions or functions on different occasions is a communicative capacity called vocal functional flexibility (Taylor et al., 2022) that occurs in cowbirds (Magdaleno et al., 2024) and humans (Oller et al., 2013) as well as possibly in related higher primates (Clay et al., 2015; Dezecache et al., 2021; Taylor et al., 2023).

The use of highly shared song types in the present study supports our hypothesis that selection favored stable song type usage due to fitness costs that would accrue if different song types were used in different contexts (Magdaleno et al., 2024) and implicates age-revealing information as having played a key role in this process by increasing the accuracy of age assessment in this species. In other words, vocal functional flexibility may have evolved in this species partly because of the increase in age-assessment provided by stable song type use across social contexts revealing repertoire size with an emphasis on shared song types, both of which indicate age. Song repertoire is not the only age indicator in male cowbirds as male ASY cowbirds tend to have more iridescent and blacker overall plumage and most SY males retain some or all of their brown juvenile underwing coverts (Selander and Giller, 1960; Ortega et al., 1996). These coverts, which are black in ASY males, are essentially hidden in the armpits most of the time but are revealed when males perform the intense wingspread displays usually done simultaneously with male-directed songs (O’Loghlen and Rothstein, 2010b). Nevertheless, none of these non-learned maturational traits are absolute in cowbirds just as is the case in humans where individuals may look younger or older than they actually are. In humans, both morphological features (e.g., looking older) and vocalizations (e.g., saying you are 38 years old and sounding older) provide information on age to recipients. So, it is likely that all available age-revealing traits and cues are assessed in species where recipients can gain reproductive benefits from this information. If age-revealing information provided by learned vocalizations improved the accuracy of age assessment, then that may have selected for the use of all song types in a male’s repertoire regardless of the context. This also raises the interesting possibility, given the importance of age for both sexes in human courtship (Conroy-Beam and Buss, 2019), that improved age assessment via learned vocalizations may have played a role in the evolution of vocal functional flexibility in humans.

One reason male-directed song displays evolved to be more intense than female-directed ones likely relates to the information important to males vs. to females. When males sing to other males, they are likely assessing each other’s current fighting ability and vigor, attributes that males can communicate via their ability to engage in long series of intense displays that accompany males countersinging with each other. In nature, it is common for 2 males (sometimes more) to each sing 12 or more songs to one another at a rate of several per minute (Rothstein et al., 1988). Song exchanges between males in nature typically end with one male (presumably the subordinate one) singing less frequently and doing less intense displays and eventually flying off or on rare occasions escalating into a physical fight. In contrast to male–male singing, the most important feature for females to assess is a male’s genetic quality, indicated more reliably by a male’s song types and repertoire size than by his current ability to engage in vigorous displays (O'Loghlen and Rothstein, 2012). Additionally, females are often present when males engage in countersinging (Rothstein et al., 1988), and this eavesdropping likely selects for presentation of a male’s entire repertoire but with overuse of widely shared song types in this context, too. Overuse of shared song types may also facilitate male’s and female’s assessment of the extent to which males match shared song types (Logue and Forstmeier, 2008).

## 5 Conclusion

The present study provides a clear example of a species that stresses the expression of learned vocalizations that signal survivorship (shared song types) and deemphasizes those expressions that do not (non-shared song types). Local song sharing is common in vocal learners (Byers et al., 2010; Otter et al., 2020; Allen et al., 2018; Schulze et al., 2022), and there are many examples of age-related differences in song characteristics and performance (Derrickson, 1987; Galeotti et al., 2001; Kochvar et al., 2022; Parapura et al., 2023), but only a few other studies have found age-related differences in the use of shared song types (playback studies, Vehrencamp et al., 2007; Kiefer et al., 2011; naturalistic observations of dawn chorus, Lapierre et al., 2011). In general, more attention needs to be given to the use of learned vocal repertoires, including the adaptive use of signals and displays that provide information about survivorship given the broad importance of age as an indicator of mate quality across taxa.

## Data Availability

The raw data supporting the conclusions of this article will be made available by the authors, without undue reservation.
